# Monitoring of Soft Deposition Layers in Liquid-Filled Tubes with Guided Acoustic Waves Excited by Clamp-on Transducers

**DOI:** 10.3390/s18020526

**Published:** 2018-02-09

**Authors:** Sabrina Tietze, Ferdinand Singer, Sandra Lasota, Sandra Ebert, Johannes Landskron, Katrin Schwuchow, Klaus Stefan Drese, Gerhard Lindner

**Affiliations:** Institute of Sensor and Actuator Technology, Coburg University of Applied Sciences and Arts, Am Hofbräuhaus 1b, 96450 Coburg, Germany; ferdinand.singer@hs-coburg.de (F.S.); sandra.lasota@hs-coburg.de (S.L.); sandra.ebert@hs-coburg.de (S.E.); johannes.landskron@hs-coburg.de (J.L.); katrin.schwuchow@hs-coburg.de (K.S.); klaus.drese@hs-coburg.de (K.S.D.); gerhard.lindner@hs-coburg.de (G.L.)

**Keywords:** biofilms, deposition layers, liquid-filled tubes, clamp-on transducers, guided acoustic waves

## Abstract

The monitoring of liquid-filled tubes with respect to the formation of soft deposition layers such as biofilms on the inner walls calls for non-invasive and long-term stable sensors, which can be attached to existing pipe structures. For this task a method is developed, which uses an ultrasonic clamp-on device. This method is based on the impact of such deposition layers on the propagation of circumferential guided waves on the pipe wall. Such waves are partly converted into longitudinal compressional waves in the liquid, which are back-converted to guided waves in a circular cross section of the pipe. Validating this approach, laboratory experiments with gelatin deposition layers on steel tubes exhibited a distinguishable sensitivity of both wave branches with respect to the thickness of such layers. This allows the monitoring of the layer growth.

## 1. Introduction

In the process industry as well as in water-supply systems soft deposition layers within liquid-filled tubes such as biogenic coatings may cause serious degradations of the process performance and health problems [1,2]. Therefore, sensors for the long-time online-monitoring of the formation of such deposition layers are highly desired [3,4,5]. Ultrasonic detection methods such as ultrasonic time-domain or frequency-domain reflectometry allow the detection of the initial phases of biofilm formation with high sensitivity [6,7], but these methods are not suited for online applications. Sensor elements based on surface acoustic waves (SAW sensors) have proven to be sufficiently sensitive for biofilm detection as well, since their displacement field is localized at the solid-liquid interface and their propagation properties are strongly affected by the formation of such layers there [8,9]. In contrast to the previously mentioned techniques, they are suited for online-monitoring, but SAW sensors have to be inserted into the tube and the information delivered is locally restricted to their insertion site, which may not be representative for the whole tube. 

In this work a non-invasive ultrasonic approach has been realized by exciting Lamb-type guided acoustic waves on the outer surface of the wall of the tube by piezoelectric transducers and utilizing changes of their transmission behaviour along the tube wall, which reflect the formation of deposition layers on the inner surface of the pipe wall [10,11,12,13,14]. However, one has to be aware that in liquid-filled tubes with metallic walls Lamb wave modes and similar wave modes with sagittal displacements at the surface experience mode conversion into compressional waves in the liquid [15,16], which limits the range of inspection of the inner wall surface. Therefore, using broad-band wedge transducers and appropriate excitation frequencies, Lohr and Rose [10] carefully selected non-leaky circumferential guided wave modes with vanishing sagittal and large in-plane displacements, which hardly suffered from mode-conversion losses in contact with water, but remained sensitive to viscous layers. This study demonstrated that proper mode and frequency selection is a crucial issue with respect to optimal sensitivity to film buildup in view of the complex wave structure of guided waves in solids [17]. In another approach, employing leaky A_0_ Lamb-type waves excited and detected with wavelength-selective piezoelectric single-phase transducers and travelling on the tube wall in axial directions, a sensitivity to adhesive tape layers on the inner surface of a water-filled plastic tube has been demonstrated [12,13].

In view of practical applications at existing tube installations, this paper shows another solution using single-phase transducers in a detachable clamp-on arrangement for circular steel tubes and circumferential leaky Lamb-type guided waves. The sensitivity to viscous deposition layers was tested with gelatin layers of different thicknesses on the inner surface of the tube. The wave modes contributing to the receiver signal were analyzed by laser Doppler vibrometry and changes of transmission signals of wave pulses with different layer thicknesses were interpreted. In addition, the ability of this method to discriminate between different types of deposition layers has been demonstrated with gelatin and lime layers and its applicability for the measurement of the filling level in horizontal pipes has been discussed. 

## 2. Materials and Methods

Following the experimental concept, the arrangement of piezoelectric transducers on a test tube, the production of gelatin deposition layers on the inner wall of the test tube, the measurement procedures and the analysis of the signals are described.

### 2.1. Experimental Concept

The experiments were performed with a tube of stainless steel V4A with nominal width DN50, an outer diameter of 60.3 mm, a wall thickness of 3 mm and a length of 300 mm. Guided circumferential waves were excited and detected on the outer surface of the wall by two piezoelectric transducers positioned exactly on opposite locations on the tube. Wave pulses of five cycles were excited by a signal generator at a frequency of 1 MHz and the receiver signals were recorded by an oscilloscope. Gelatin layers of different thicknesses and layers of lime were deposited on the inner surface of the test tube, which subsequently was filled with water and the corresponding transit times and amplitudes of the receiver signals were analyzed. In addition, the influence of temperature changes on the receiver signals and the influence of incomplete filling levels in horizontal tubes were investigated.

### 2.2. Transducer Arrangement

For the excitation and detection of surface acoustic waves on the tube wall single-phase transducers made of PZT (type PIC255, obtained from PICeramic GmbH, Lederhose, Germany) were used. These wavelength-selective thin transducers (6 mm × 4 mm × 1 mm) have a two-finger electrode structure at the bottom to excite plane surface acoustic waves perpendicular to the electrode fingers with a wavelength of 3 mm on the underlying solid [13,18]. When driven at a frequency of 1 MHz different from a vibrational resonance of the transducer, this wavelength corresponds to an A_0_ Lamb wave on a 3 mm thick steel plate travelling with a phase velocity of almost 3000 m/s. The orientation of the transducers was chosen such that they excite plane guided waves predominantly travelling circumferentially in both directions around the tube, i.e., the finger electrodes were oriented parallel to an axial direction. The receiver position exactly opposite to the transmitter ensured that both wave pulses arrive at the receiver at the same time. This is accomplished by precision manufacturing of both yokes using a CNC milling machine. In addition, the screws on the left and the right side of the tube fix the two parts of the clamp-on device at the tube in exactly equidistant positions.

**Figure 1:**
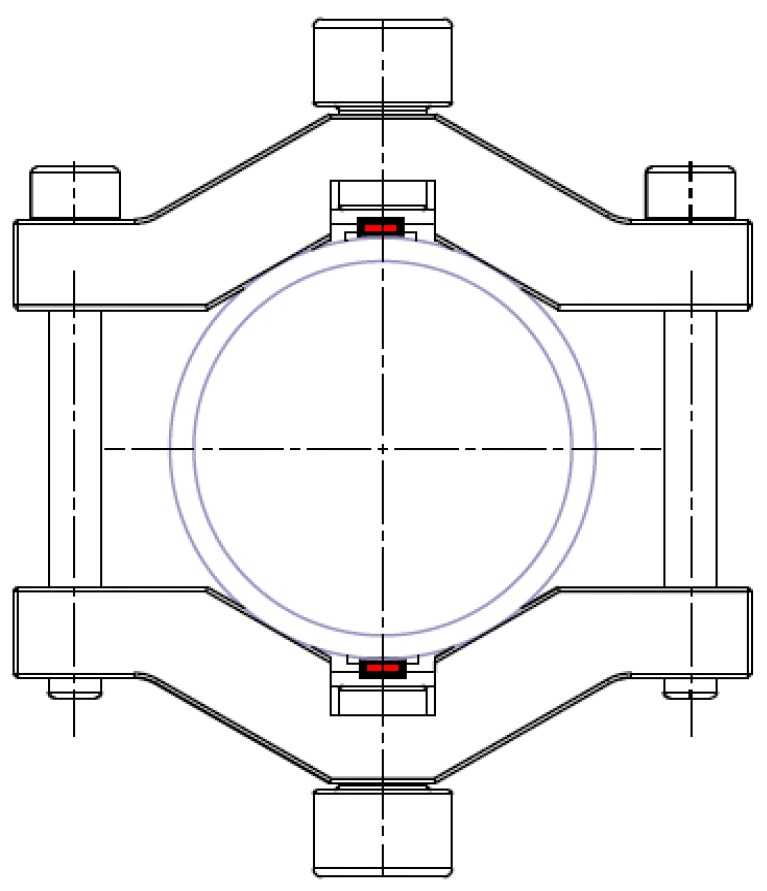
Cross-sectional drawing of the tube with clamp-on device (**black**) and transducer (**red**). The screws left and right of the tube fix the clamping yoke to the tube (**blue**); the screws on top and bottom press the piezoelectric transducers against the tube wall.

In order to facilitate the proper orientation of the transducers in the clamp-on construction, the transducers were soldered to a rectangular board, which fits into a corresponding slot in the center of the clamp. The transducers were pressed against the tube wall by a screw directly above the transducer board. For better mechanical stability, a thin (1 mm) soft PVC mat with a width of 7 mm is glued to the outer tube wall below the transducer with a two component epoxy glue to ensure stability and a uniform mechanical contact free of bending stress between the transducer and the tube wall. In addition, a thin layer of an ultrasound coupling compound (AntiSize, WEICON GmbH & Co. KG, Münster, Germany) between the transducer and the PVC mat ensures a large sound transmission. A schematic drawing of the clamp-on device and the transducer arrangement is shown in Figure 1. 

### 2.3. Sample Preparation

Gelatin layers were deposited on the inner wall of the tube for testing the sensitivity of this acoustic device for the detection of soft layers. Different thicknesses of the deposition layers were obtained by changing the concentration of gelatin in solution. To achieve a good reproducibility of the gelatin layers and a good adhesion to the wall, glycerol was added to the gelatin solution. Starting with a 1:1 water/glycerol mixture of 500 mL, five different amounts of gelatin (25, 50, 75, 100, 125 g) were added to obtain five different layer thicknesses. The tube was filled with the mixture of water, glycerol and gelatin at 65 °C for twenty seconds. Afterwards, the tube was rotated upside down to remove the mixture and the remaining liquid was drained out of the tube for ten minutes. Subsequently, the tube was filled with water of room temperature and the measurement was started. Finally the tube was emptied and cleaned and the procedure was repeated subsequently with increasing gelatin concentrations.

For the determination of the layer thicknesses, a stainless steel plate was immersed into the water-glycerol-gelatin mixtures and run off for ten minutes together with the tube measurements. Half of the plate was covered with sticky tape, which was removed from the plate after drying. In this way, an edge of the gelatin layer was produced, whose height was measured with a laser scanning microscope (VKX200, KEYENCE DEUTSCHLAND GmbH, Neu-Isenburg, Germany) after immersing the plates into petri dishes filled with water at room temperature. It was assumed that the measured height of the edge is a reasonable estimate of the gelatin layer thickness in the tube, although the latter might be a little bit thicker because of the curvature of the tube. By repeating the layer thickness measurement with each gelatin concentration five times for each gelatin concentration, a calibration relationship between the layer thickness and the gelatin concentration with standard deviations of the layer thickness of less than 10% for each concentration value was obtained. This good reproducibility was considered as an indication for the formation of rather homogeneous gelatin layers in the tube as well. Small local inhomogenities are rather negligible in view of the pitch-catch measurement concept, which averages across thickness variations along the propagation path of the wave pulses.

### 2.4. Measurement Devices and Procedures

The transmitter was driven by a function generator (33220A, Keysight Technologies Deutschland GmbH, Boeblingen, Germany) with a five-cycle sine tone burst at 20 V_pp_ and a frequency of 1 MHz. The receiver signals were recorded with a digital storage oscilloscope (WaveRunner 604Zi, Teledyne LeCroy GmbH, Heidelberg, Germany). The transmission time of such a wave burst was measured with respect to a zero crossing of the corresponding signal near the cycle with the maximum amplitude. At the same receiver signal, the maximum peak-to-peak value of a cycle was measured as well. With the integrated functions of the oscilloscope, the transmission time and the peak-to-peak amplitude were averaged over 300 values. After completion of a measurement series with an empty tube and five different gelatin layers, the clamp-on device was removed from the tube. Finally, the recorded transmission times and amplitudes were related to the initial values with the water-filled tube without gelatin layer; i.e., the corresponding changes in time and amplitude were determined. The whole experimental procedure was repeated three times with increasingly thicker gelatin layers and the mean values and standard deviations from the respective changes of transmission times and amplitudes were calculated.

## 3. Results

### 3.1. Acoustic Wave Pulse Propagation in a Water Filled Test Tube

In order to provide a sound baseline for investigations of the influence of deposition layers on the propagation of acoustic wave pulses, a thorough characterization of the wave pulse propagation in empty and water filled test tubes was necessary. To this end, numerical simulations, scanning laser Doppler vibrometer measurements both on the tube wall and within a water-filled tube and an analysis of the receiver signals obtained from burst excitation of wave pulses on the tube wall were performed. 

#### 3.1.1. Numerical Simulations

**Figure 2:**
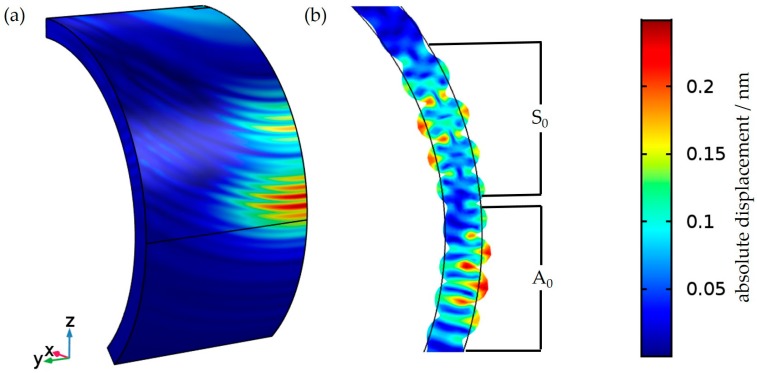
COMSOL simulation of (**a**) the propagation of a wave pulse on the tube wall excited with a single-phase transducer; (**b**) Cross-sectional view of the wave propagation across the tube wall indicating S_0_ and A_0_ Lamb-type modes.

A numerical simulation of an acoustic wave pulse excited by a single-phase transducer travelling along the empty steel tube wall in circumferential direction was calculated with the structural mechanics module of COMSOL Multiphysics 5.3.0.248 (Comsol Multiphysics GmbH, Berlin, Germany). Because of the symmetric conditions, just a quarter segment of the pipe and the transducer needs to be implemented in the model (Figure 2). To simulate the vibration behaviour of the transducer, the experimental well-known vibration behaviour of the transducer was implemented as boundary condition. The excitation of the transducer was located at the top right corner of the picture (simulated part of the transducer: length 3 mm, width 2 mm). The upper and right intersections are constrained with symmetric boundary conditions to represent the remaining pipe. To avoid reflections on the left and the lower ending of the simulations area, these intersections are coated by “low-reflection boundary” conditions. A structural net with a maximum edge length of 0.25 mm was used. The equations are solved in time domain with a step width of 50 ns per iteration.

The results of the simulation are visualized in Figure 2. Two different wave packets can be observed. The cross-sectional view of the wave propagation at the tube wall indicate an A_0_ and a S_0_ Lamb-type mode because of the antisymmetric and symmetric displacements, respectively (Figure 2b). A wavelength of 3 mm was obtained at an excitation frequency of 1 MHz at the first wave packet. Although there is some bending of the wave packets in axial direction, the largest amplitudes appear along a circumferential pathway and the wavelength and the propagation velocity of the first wave packet are similar to that of an A_0_ Lamb wave on a plane plate having the same height as the wall thickness. Therefore, in view of the purpose of this paper, it seems to be justified to neglect the bending of the tube wall in that respect and to regard the wave motion in circumferential direction in terms of Lamb waves on plane plates. This includes the replacement of the correct denomination “lowest order flexural Lamb-like wave (A_0_ mode)” by “A_0_ Lamb-type mode” or simply “Lamb-type guided wave” in the following [17]. The second wave packet, which is indicating a S_0_ Lamb-type mode, will be neglected in the following because of its weak excitation in the system investigated. 

#### 3.1.2. Visualization of the Acoustic Wave Propagation with a Scanning Laser Doppler Vibrometer

**Figure 3:**
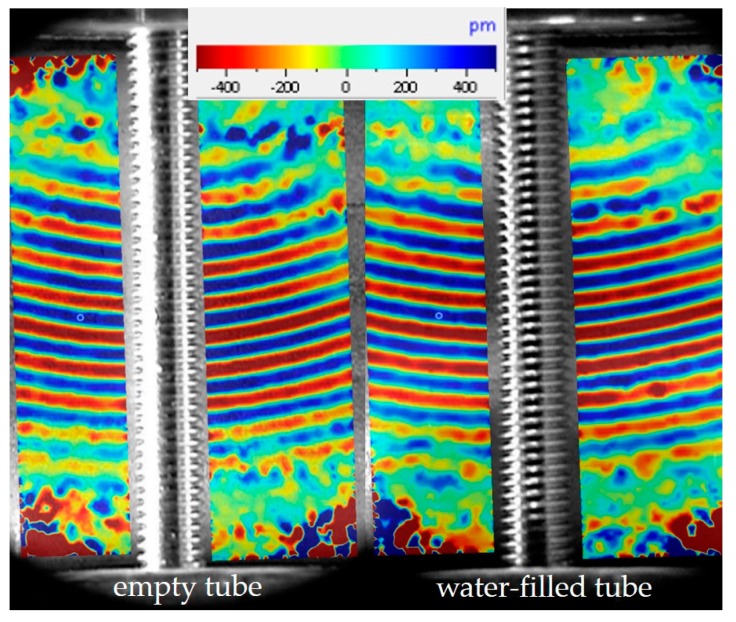
SLDV record of an acoustic wave pulse travelling on the wall of an empty (**left part**) and a water filled (**right part**) test tube. The colors show the vertical displacement in pm at the time of 30.57 µs after pulse generation.

The numerical simulations of the wave propagation on the tube wall were validated by scanning laser Doppler vibrometer (SLDV) recordings of wave pulses on the wall of an empty test tube (Figure 3 left part). For this purpose, a commercial SLDV instrument (PSV 400M, Polytec GmbH, Waldbronn, Germany) was used. For comparison, the wave propagation on the wall of a water-filled test tube was recorded by SLDV as well (Figure 3, right part). The scan ranged over the tube wall between the two yokes with the scanning laser beam impinging from the right side of the tube in an axial view as in Figure 1; i.e., the connecting screws visible in Figure 3 are oriented vertically. The exciting transducer was on top outside the range of the figure and the waves were travelling downwards. Obviously, there is a high similarity of the shapes and the wavelength of the apparent wave mode with the numerical simulations shown in Figure 2. Since the SLDV is only sensitive to displacements of the wall surface in the direction of the scanning laser beam, the observations corroborate the attribution to the antisymmetric A_0_ mode of a Lamb wave because of its prominent vertical displacements and its wavelength [17].

This attribution is justified also by experiences with circumferential waves from other authors: as long as the ratio between inner and outer radius of the tube is near to one, i.e., the wall thickness is small compared to the inner and outer radius, the dispersion curves of the guided circumferential waves are very similar to those of Lamb waves on a flat plate of the same thickness [19,20,21]. In our case, the ratio of inner and outer radius is 0.9, which is close to the values for which no significant differences to the dispersion curves of Lamb waves were reported [19]. Other research groups report that mode conversion effects of corresponding circumferential waves were observed in contact with water in the same way as with Lamb waves [22,23]. These findings further support the appellation of the dominating mode as “A_0_ Lamb-type mode” as indicated in Section 3.1.1.

**Figure 4:**
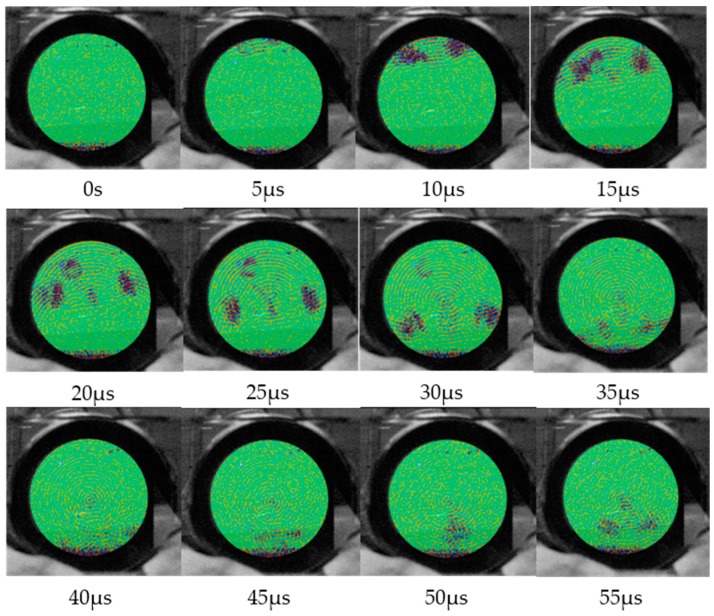
SLDV records of an acoustic wave pulse transmitting the cross-section of a water-filled test tube at different instants of time. Between t = 5 µs and t = 35 µs, a compressional wave is radiated under the Lamb angle in mirror symmetric manner into the liquid (mode conversion); between t = 40 µs and t = 55 µs, the mode-converted wave arrives at the tube wall, where it is partially reconverted into an A_0_ Lamb-type mode on the tube wall as well as specularly back-reflected into the liquid.

Additionally, the propagation of sound wave pulses within the water-filled test tube was visualized by axial SLDV scans as shown in Figure 4. 

Thereby the tube was placed horizontally in a rectangular water-filled glass vessel. The tube was immersed in water, so that the inner of the tube was completely filled with water but the transducer on top of the tube remained slightly above the liquid level. Thus, the wave is generated outside of the water and propagate along the tube wall. The laser beam of the SLDV was focused at the back wall of the vessel, allowing the recording of pressure changes in the liquid caused by acoustic waves. Since the A_0_ Lamb-type mode phase velocity on the wall *c_L,ph_* is larger than the velocity of sound in water *c_W_*, mode conversion occurs and a wave pulse is radiated into the water at an angle according to Snells law of refraction:

(1) $$\theta_{L}=\arcsin\left(c_{W}/c_{L,ph}\right).$$

Because *θ_L_* is determined by the phase velocity of the Lamb wave, *θ_L_* is called Lamb angle in the following (see scan record at 20 µs in Figure 4). Since the single-phase transducer excites circumferential waves in opposite directions, the radiation occurs twice in mirror symmetric manner. In addition to the angular mode conversion radiation, there is a weak signal from a longitudinal wave pulse travelling straightly in vertical direction, which may result from a thickness vibration component of the transducer deformation. The mode conversion radiation eventually arrives at the tube wall again (see scan record at 40 µs in Figure 4), where it is partially reconverted into an A_0_ Lamb-type mode, which travels to the receiver transducer. Both specular reflections and back conversion happen afterwards (see scan records after 40 µs in Figure 4), which however no longer affect the receiver signal. 

The SLDV records prove that both circumferential A_0_ Lamb-type waves and compressional waves transmitted across the water-filling contribute to the receiver signal. Due to their different velocities they will arrive at the receiver transducer at different instants of time.

#### 3.1.3. Analysis of Receiver Signals from Acoustic Wave Pulses Obtained at the Test Tube

**Figure 5:**
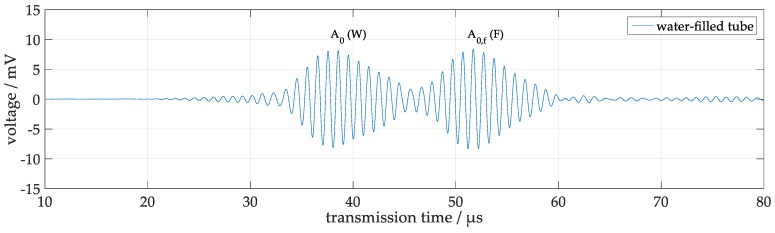
Receiver signal from a single excitation burst measured at a water-filled tube, exhibiting two components assigned to an A_0_ Lamb-type mode on the tube wall centered around 38 µs (W) and to a compressional wave crossing the water resulting from mode conversion A_0f_ centered around 52 µs (F).

The measured receiver signals obtained at a water-filled steel test tube after a burst excitation with 5 cycles and a frequency of 1 MHz are displayed in Figure 5. Obviously, there are two distinct prominent components centered around 38 µs (component W) and 52 µs (component F). According to the SLDV observations, these components can tentatively be attributed to the A_0_ Lamb-type mode along the tube wall and the mode-converted compressional wave across the water A_0f_, respectively (Figure 5). It should be kept in mind that due to the symmetric emission of two wave pulses from the exciting transducer in opposite directions, the shape of the signals emerges from the superposition of both wave branches and will allow a simple evaluation only if the transducers are located on exactly opposite positions. 

**Figure 6:**
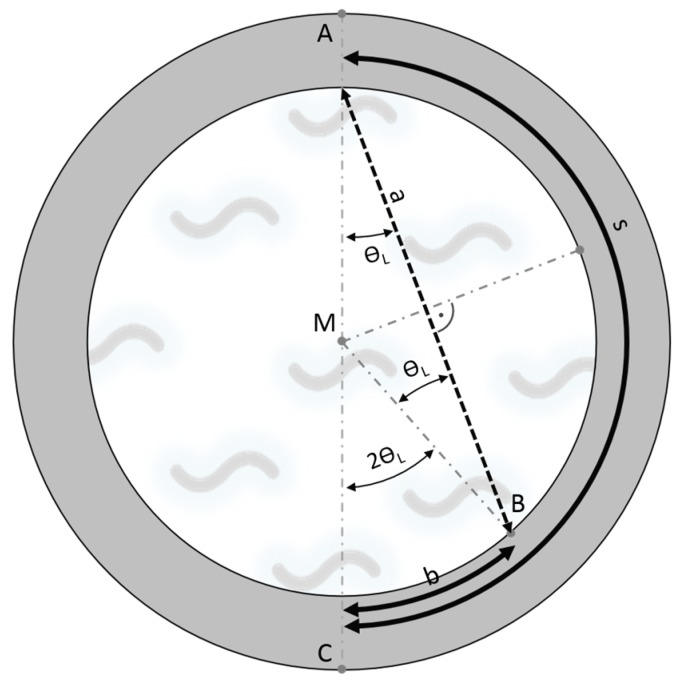
Geometrical explanation of transmission pathways of acoustic wave pulses in the water-filled tube: wave pulse along the tube wall travelling from A to C and mode-converted compressional wave pulse in the water from A to B, which is reconverted into a wave pulse travelling from B to C.

This interpretation is corroborated by calculations of the transmission times from the lengths of the travelling pathways of the two wave packets and the corresponding propagation velocities. The geometric relationships used for the calculation of the simulated signal for the pathways are outlined in Figure 6. 

The Lamb-type wave, which is not mode-converted, travels a distance of:

(2) s=π×r,

along the tube wall from point A to C, with *r* tube diameter. The Lamb-type wave which is mode-converted into a compressional wave travels a pathlength *a* through the liquid:

(3) $$a=2\times r\times \cos\theta_{L}.$$

The mode-converted wave is then reversely mode-converted into a Lamb-type wave at point B and travels the remaining distance *b* along the tube wall:

(4) $$b=2\times \theta_{L}\times r.$$

Only the wave propagation pathways in the right part of tube cross-section are depicted; the pathways in the left half are mirror-inverted identically. 

**Figure 7:**
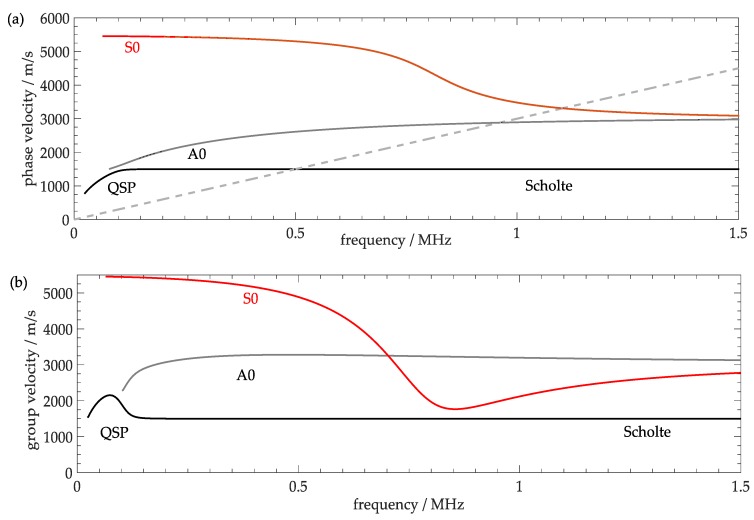
Calculated dispersion diagrams, i.e., phase velocity (**a**) and group velocity (**b**) as a function of frequency for Lamb and Scholte waves on steel plates with a thickness of 3 mm covered with water on one side using the software package “DISPERSE” together with the excitation line (grey dashed) of the wavelength-selective single-phase transducer in (**a**).

The A_0_ Lamb-type mode phase and group velocities *c_Lph_* and *c_Lgr_* were obtained from a calculated dispersion diagram for corresponding Lamb waves displayed in Figure 7, for which the software package DISPERSE (Version 2.0.20a, IC Consultants Ltd, London, Great Britain) was used. Additionally, the excitation line for the wavelength-selective single-phase transducer is included in the phase velocity diagram, which indicates the frequencies, at which certain modes can be excited by the crossing points with the respective dispersion curves [17]. With the values *c_W_* = 1480 m/s, *c_l,steel_* = 5960 m/s, *c_t,steel_* = 3260 m/s, *ρ_steel_* = 7.932 g/cm³, and *r* = 29.4 mm, a Lamb angle of 30.77° was calculated.

In order to estimate the arrival times of peak intensities, group velocities *c_L,gr_* are used for calculation according to Figure 7b. The arrival time of the Lamb-wave pulse which remains on the tube wall is:

(5) $$t_{1}=\frac{s}{c_{L,gr}}=\frac{\pi \times r}{c_{L,gr}}.$$

The arrival time of the double mode-converted pulse is:

(6) $$t_{2}=\frac{a}{c_{w}}+\frac{b}{c_{L,gr}}=2\times r\times \left(\frac{cos\theta_{L}}{c_{w}}+\frac{\theta_{L}}{c_{L,gr}}\right).$$

**Figure 8:**
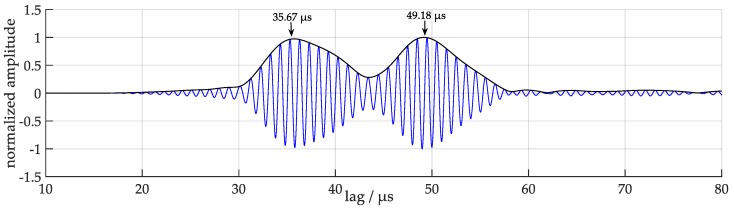
Cross-correlation of a Hanning windowed 5-cycle sine wave of 1 MHz with the measured signal together with the resulting arrival times.

With these formulas values of *t*_1_ = 28.90 µs and *t*_2_ = 44.02 µs are calculated. For a comparison with measured values the influence of the 1 mm thick soft PVC mat has to be taken into account additionally. Since the material parameters of the mat are not exactly known, a corresponding transmission time of 5 µs was extracted empirically from the regression line used for the determination of the sound velocity in water from the liquid level measurements described in Section 3.4. This results in effective arrival times of *t_1eff_* = 33.90 µs and *t_2eff_* = 49.02 µs. Measured values of arrival times were obtained with the cross correlation method: the transmitted signal was simulated by a Hanning windowed 5-cycle sine wave, which takes into account the vibrational behaviour of the transducer. This signal is cross-correlated with the measured signal resulting in arrival time values of *t*_1_ = 35.67 µs and *t*_2_ = 49.18 µs (Figure 8), which reasonably agree with the calculated values given above. The small differences are attributed to uncertainties of the material parameters and the time delay caused by the soft PVC mat.

**Figure 9:**
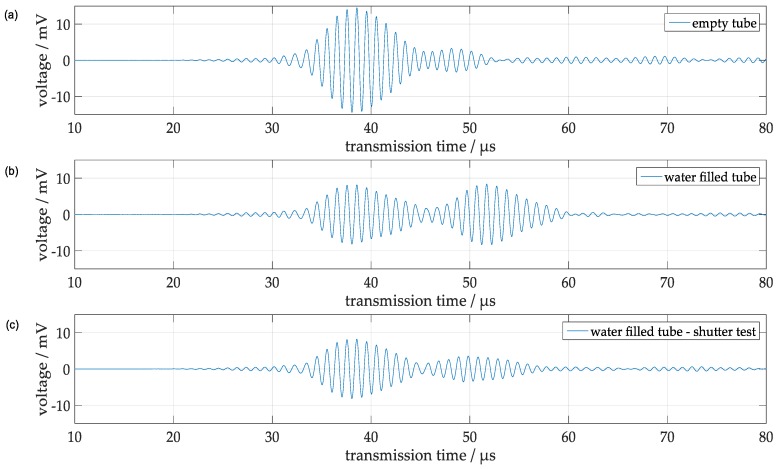
Signals obtained at a clean steel test tube at 1 MHz (**a**) empty tube; (**b**) water-filled tube; (**c**) water-filled tube and with a shutter in the water.

Another corroboration of the interpretation of the two prominent components in the receiver signal of Figure 5 is obtained by comparing the signals measured (a) at an empty tube, (b) at a water-filled tube and (c) at a water-filled tube with a shutter blocking the sound transmission through the water (Figure 9). Obviously, at the empty tube the signal from the A_0_ Lamb wave is most prominent (Figure 9a), whereas at the water filled tube, the amplitude of this signal is reduced and a second wave packet, which is attributed to a pathway of compressional waves from mode conversion through the water, appears (Figure 9b). With a shutter blocking that pathway, this second wave packet vanishes as expected (Figure 9c). Although of minor relevance for the following results, the small signals centered around 48 µs in Figure 9a and around 50 µs in Figure 9c deserve some consideration. The small second wave packet in Figure 9a results from the excitation of a S_0_-mode having a group velocity smaller than that of the A_0_ mode at 1 MHz excitation frequency (see Figure 7b). Unfortunately, this signal could not be eliminated completely and it will also contribute to the signal of the water-filled tube (Figure 9b) and interfere to some extent with the signal from mode-converted waves. On the other hand, the influence on the parameters of that signal, which will be used for quantitative evaluations, i.e., the maximum cycle amplitude and the time of zero crossing adjacent to the cycle with the largest amplitude, will be negligible. This is because the cycle amplitude of the S_0_ signal at this instant of time near 52 µs is already smaller than 1 mV in the empty tube and will further decrease in the water-filled tube due to mode conversion. The second wave packet in Figure 9c centered around 50 µs may represent parts of the mode-converted waves, which are not completely blocked by the shutter which consists of a plastic shutter with a width of 30 mm inserted axially into the tube within a plane perpendicular to the connecting line between the transducers. Since its width is smaller than the inner diameter, only a part of the mode-converted wave was blocked and the corresponding signal represents the remainder of that wave, which could pass in the interstice or which was reflected by the edge of the shutter. 

#### 3.1.4. Influence of Temperature

**Figure 10:**
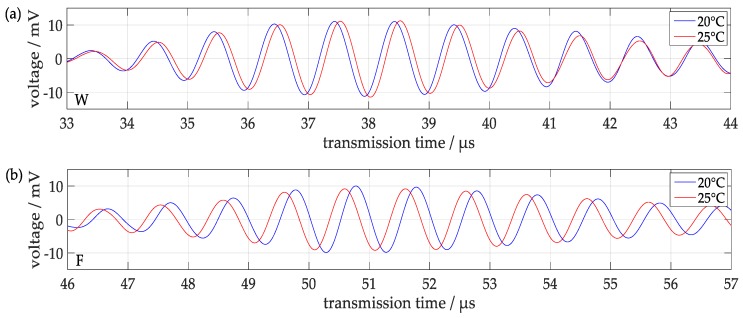
Signals obtained from a clean water-filled tube at different temperatures around room temperature (**a**) first signal component W; (**b**) second signal component F.

The influence of temperature, which is always crucial for acoustic measurement concepts, and results from the temperature dependence of the sound velocity and the sound attenuation in different media, has thoroughly been investigated by separate measurement series. Signals measured at a water-filled tube at temperatures between 20 °C and 25 °C, i.e., within a temperature range of 5 °C, are displayed in Figure 10. There were small monotonous changes both of the transmission times and the amplitudes detectable, which meet the expectations from known temperature dependencies in the materials used [24,25]: the large positive temperature coefficient of the sound velocity in water (+1.94 × 10^−3^ °C^−1^ at 20 °C) and the small negative temperature coefficient of Rayleigh waves in steel (−2.1 × 10^−4^ °C^−1^ at 20 °C) are expected to shift the transmission time of the component W to larger and of the component F to shorter values, which agrees with the observations in Figure 10. According to the relationships between transmission times and wave velocities changes of the transmission times of +0.03 µs of component W and of −0.31 µs of component F were predicted, which fit with respect to sign and order of magnitude to the measured time shifts of +0.08 µs of component W and −0.20 µs of component F. With a more sophisticated temperature calibration, it seems possible to compensate influences of temperature on the measurements in future.

### 3.2. Influence of Gelatin Layers

**Figure 11:**
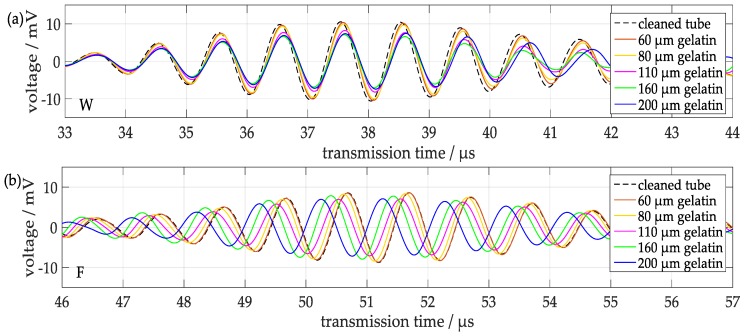
Signals from a test tube with gelatin layers with different thicknesses: (**a**) first signal component W; (**b**) second signal component F.

**Figure 12:**
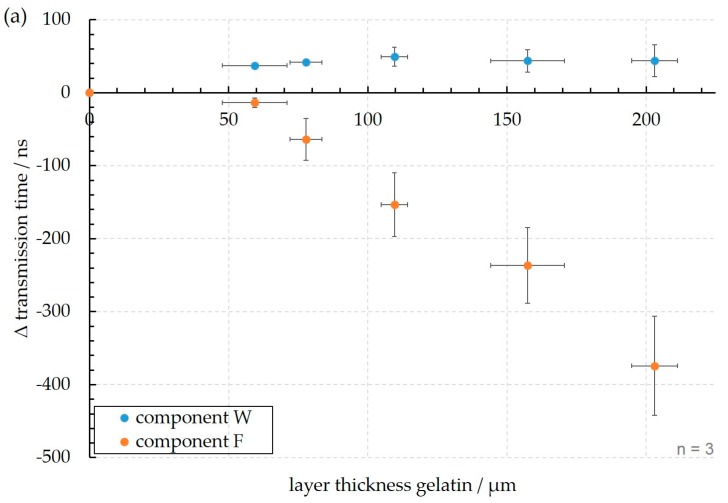

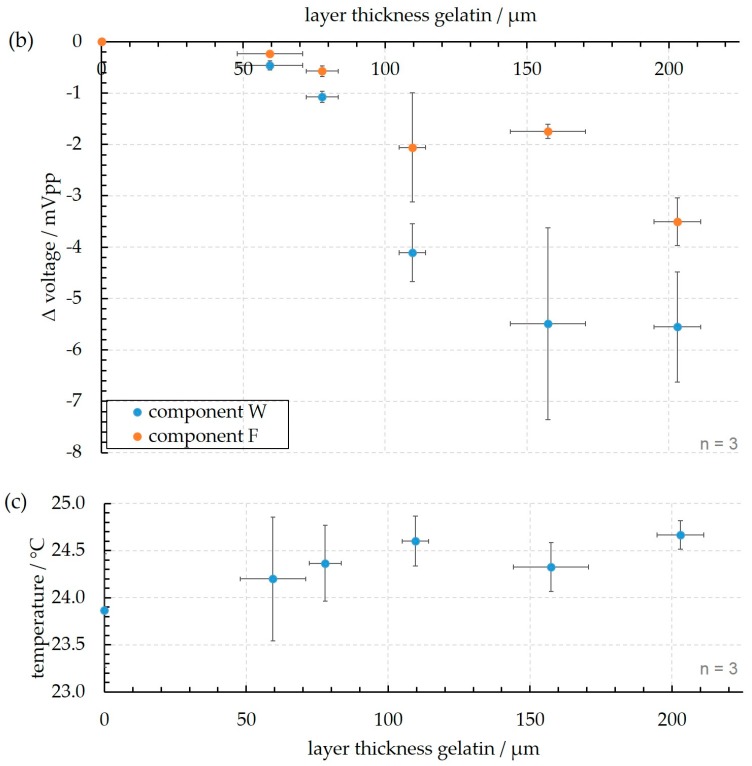
Changes of the transmission parameters of both signal components in dependency of the gelatin layer thickness (**a**) transmission time; (**b**) amplitudes of received signals and (**c**) temperature during the measurements. The measurements are repeated three times (*n* = 3).

In order to investigate the sensitivity of this device with respect to soft layers on the inner wall of the tube, layers of gelatin with different thicknesses were subsequently deposited according to the procedure described in Section 2.3. The signals of transmitted wave pulses were recorded and analyzed with respect to the transmission times and the maximum peak-to-peak amplitudes. As shown in Figure 11 and Figure 12, the signals of both components exhibit a decrease of the amplitudes with increasing thickness of the gelatin layer and shifts of the transmission times, which, however, are rather small and towards longer times for component W, whereas for component F they are more prominent and towards shorter transmission times. 

### 3.3. Influence of Other Deposition Layers

**Figure 13:**
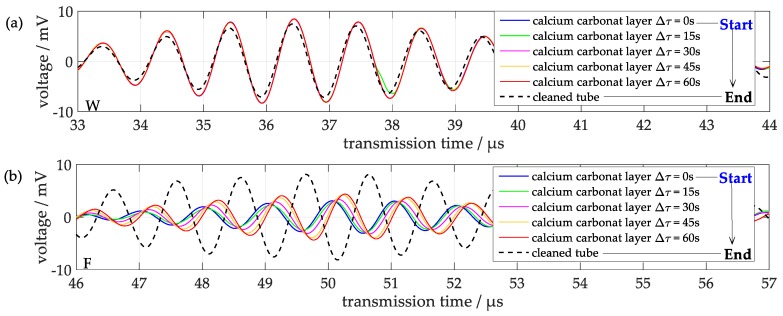
Both signal components before deposition of a calcium carbonate layer, after deposition and after subsequent partial dissolution of the deposition layer. (**a**) signal component W, (**b**), signal component F.

Concerning practical applications, however, it is often desirable to identify the species of a deposition layer, in particular to discriminate between a hard layer such as limescale or furring and a soft layer such as a biofilm. In experiments with a test tube, a calcium carbonate layer of initially about 1 mm thickness was deposited first and then was subsequently dissolved again in the course of time, i.e., the thickness decreased monotonously. Unfortunately, the thickness of the remaining layers could not be measured directly; therefore the dissolution time *Δτ* serves as an indicator of the amount of reduction of the layer thickness. Figure 13 shows corresponding signals, which demonstrate that the signal changes compared to the bare tube both with respect to time shifts and the attenuations are significantly different from those observed with gelatin layers. There is only a small change in transmission time and attenuation at the component W as it can be seen in Figure 13a, whereas component F in Figure 13b shows a large change in transmission time and attenuation by the calcium carbonate layer. That means, initially after the calcium carbonate deposition, when the layer is thickest, a large time shift of component F of about 0.6 µs to smaller transmission times was observed, which was partially compensated afterwards due to the decrease of the layer thickness with increasing dissolution time. A similar behaviour of the signal amplitudes of component F was observed. In contrast, the corresponding time shifts and amplitude changes of component W are significantly different, since both the transmission times and the amplitudes are larger with the calcium carbonate layer. The pattern of changes allows a discrimination from the effect of gelatin layers (Figure 12), in which the amplitudes of component W are decreasing as well with increasing layer thickness, and thus enables to distinguish between hard and soft deposition layers in applications at tubes with unknown types of depositions.

### 3.4. Influence of Filling Level in Horizontal Tubes

**Figure 14:**
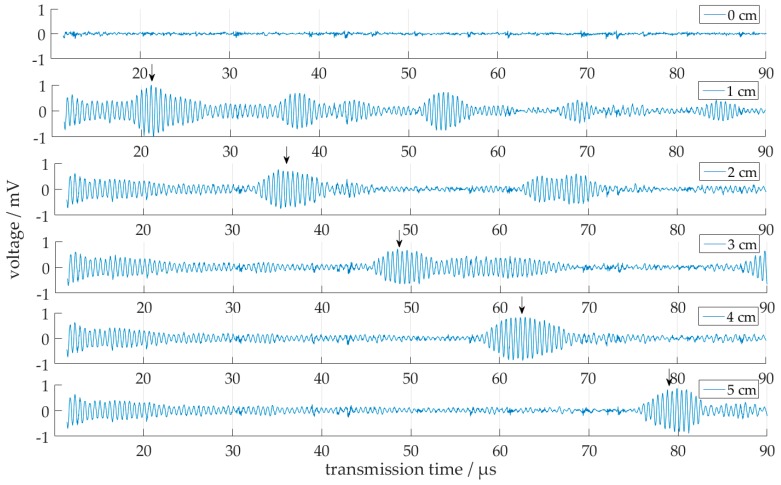
Signals at 1.875 MHz from a partially filled horizontal tube after subtraction of a reference signal obtained at an empty tube. The arrows indicate the echo pulses from the water surface; for each signal row the corresponding filling level in cm is indicated in the right upper corner.

Another demand from practical applications may be the determination of the water filling level in partially filled horizontal tubes. In this case a two-frequency approach is advisable: at the thickness vibration resonance of the transducer at 1.875 MHz the level can be measured with the well-known pulse echo technique driving the lower transducer alone. In Figure 14, such echo signals are displayed for different water levels of 0 to 5 cm in a horizontal test tube. A reference signal from an empty tube has been subtracted in order to enhance the signal to noise ratio for the echo signal. It could be observed that with increasing filling level the wave packet shift turned to right. The received signal is the reflected signal from the water surface, because the sound wave is transmitted from the bottom of the tube. Thereby with an increasing filling level the propagation path of the sound waves is getting longer and results in a higher transmission time. As a coarse check of this concept, the sound velocity *c_W_* of water was calculated from the measured pulse transmission times (indicated by arrows in Figure 14). Thereby the difference of the transmission time between the different filling levels is approximately 14 µs. This results in a calculated sound velocity of water of 1430 m/s. This value is smaller than the literature value of 1483 m/s at 20 °C, but this deviation is within the uncertainties from the level determination, the material parameters and the water temperature, which has not been measured in this rough test. 

## 4. Discussion

**Figure 15:**
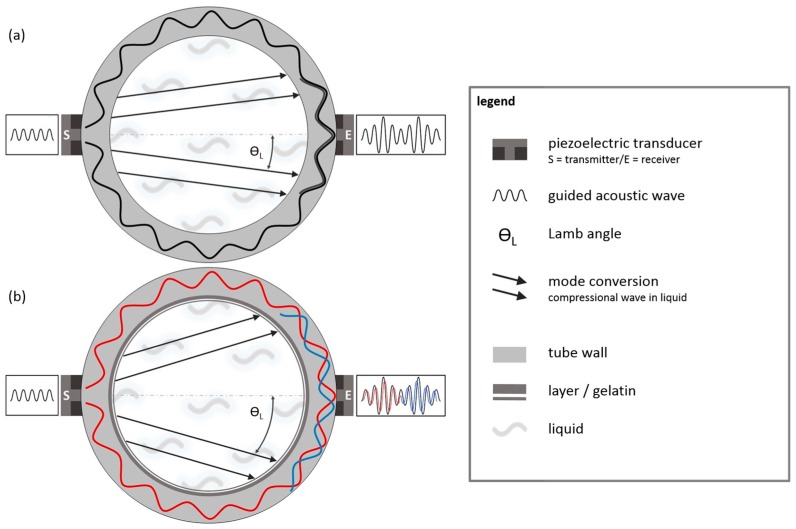
Proposed interpretation of the observed signal changes due to gelatin layers. (**a**) signals without deposition layer (black); (**b**) signals with a gelatin deposition layer (red—component W, blue—component F).

The changes of transit times and amplitudes observed with gelatin layers are explained in the following within the framework of a simple geometrical approach (Figure 15). The differences observed in the signal changes caused by soft and hard deposition layers are discussed with respect to the possibility of a discrimination between different layer types. Additionally, the capability of this device for filling level measurements at partially filled horizontal pipes is judged. 

In all following interpretations, a geometrical approach comparable with geometric optics is used for the reconstruction of the propagation pathways of the sound waves inside the tube. In particular with respect to the acoustic radiation from leaky circumferential guided waves, the propagation pathway is represented by the movement of the initial edge of the compressional wave in the liquid, since this will determine the transmission time and the amplitude of the corresponding receiver signal. 

### 4.1. Signal Changes Resulting from Gelatin Layers

Based on the interpretation of the components W and F in terms of A_0_ Lamb-type guided waves and compressional waves through the liquid, respectively, explanations for the observed time shifts and amplitude changes are proposed. Considering the gelatin layer to be a viscoelastic layer, a larger damping of both wave packages compared to pure water and steel as media of sound transmission was to be expected [11,26,27], which explains the amplitude reduction observed in both cases. The retardation of the A_0_ Lamb-type mode on the tube wall (component W) due to a viscoelastic layer is understandable as well from previous investigations [12,13,27,28] and from investigations with similar types of surface acoustic waves. With circumferential shear-horizontal type waves for example, Rose [17] reported a reduction of the velocity on a steel tube of 0.3% at a layer thickness of 180 µm, when a viscoelastic bitumenous layer was applied on the outer wall surface. In former own measurements with Lamb waves on thin plates, when a soft layer was deposited (e.g., gelatin on glass or adhesive tape on steel), reductions of the Lamb wave velocity in the order of 0.5% were measured for a layer thickness of 200 µm [12,13]. This explains why only small and positive changes of the transmission time of component W were observed (Figure 12a). As outlined by Vellekoop [29], the mass increase resulting from the deposition of gelatin may contribute to the velocity change of component W. According to the formulas of the mass sensitivity of the Lamb wave velocity given therein, however, only a very small change of the velocity below 0.1% is expected for the system investigated in this study. This is mainly caused by the large thickness of the tube wall compared to the deposition layer in contrast to Lamb wave devices using flexural waves on very thin membranes (2 to 4 µm) covered with gel layers of 250 µm and which are operated at frequencies in the order of several MHz [28]. 

With respect to the time changes of component F, the influence of the mass increase on the Lamb angle can also be neglected compared to the influence of the viscoelastic properties of the gelatin layer as explained in the following. The observed decrease of the transmission time of component F with increasing layer thickness is intriguing at the first glimpse. The transmission time of component F, however, partially depends on the pathlength of the mode-converted wave in the water (distance a in Figure 6), which is determined by the Lamb angle *θ_L_*. This angle depends on the phase velocities of the modes propagating at the interface between the gelatin layer and the water. As discussed by Simonetti [26] in his investigations on the propagation of Lamb waves in a bilayer system consisting of metallic plates coated with viscoelastic layers, modes travelling primarily in the elastic plate or remaining trapped in the viscoelastic layer can be distinguished. In the corresponding dispersion diagrams, the existence of such mode splitting becomes apparent by a splitting of the original phase velocity function of the mode equivalent to an A_0_ mode into two branches above a certain frequency, where the upper branch with the larger phase velocity represents a fast mode in the elastic plate and the lower branch a slow mode in the viscoelastic layer (see e.g., Figure 5 in [26]).

In our case, we assumed that such a mode splitting also occurs in the steel/gelatin bilayer system and that the slow mode contributes to sound radiation into the water with a Lamb angle, which is determined by its smaller phase velocity. This angle is larger than the Lamb angle of the fast mode and is therefore responsible for the shortening of the transmission time of the component F. In order to corroborate this assumption, we performed phase velocity dispersion calculations using DISPERSE for a model system consisting of a steel plate covered with a thin gelatin layer assuming estimated material parameters for the gelatin. A corresponding mode splitting was obtained and the bifurcation frequency shifted to smaller frequencies with increasing gelatin layer thicknesses, resulting in a decreasing phase velocity of the slower mode. Since the material parameters of our gelatin layers, in particular the storage modulus (which strongly depends on the gelatin concentration [30]) are not exactly known, a quantitative calculation of the dispersion function of the slow mode and a comparison of corresponding Lamb angles and resulting transmission times with measured values is not possible up to now. On the other hand, a simple model based on the assumption of the existence of such a slower mode and a decrease of its phase velocity with increasing layer thickness may provide an indication that this approach is promising (Figure 15). Assuming a phase velocity of the slower mode, which is monotonously decreasing with layer thickness, and a correspondingly increasing Lamb angle and a constant phase velocity of the faster mode on the steel wall, the calculated transmission time of component F decreases monotonously with layer thickness. 

### 4.2. Signal Changes Resulting from Hard Deposition Layers

**Figure 16:**
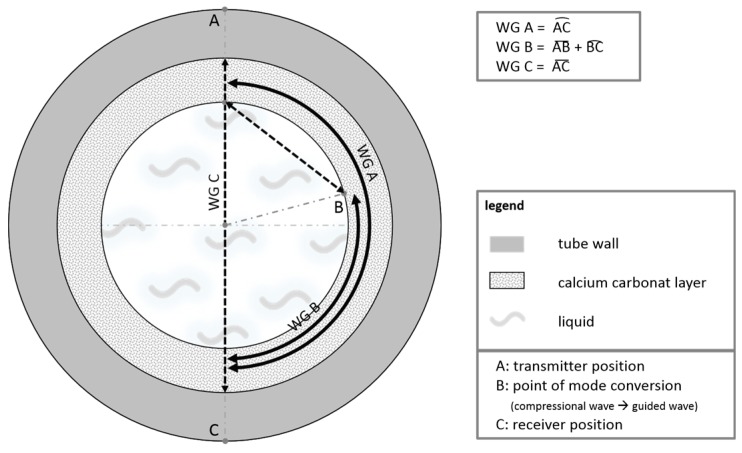
Explanation of three different pathways of sound waves in a water-filled tube with a solid deposition layer (light grey layer) on the inner wall surface.

The observed strong impact of a hard deposition layer on the transmission times can be explained by geometrical considerations as outlined in Figure 16: when the inner diameter shrinks, the pathway length of the compressional wave in the water resulting from mode conversion decreases and correspondingly the transmission time of component F decreases and approaches more and more the transmission time of component W on the tube wall. The attenuation of the sound waves, on the other hand, is smaller for component W than in the case of a soft gelatin layer, but larger for component F, which indicates that the mode conversion is sensitively disturbed by this type of layer. Nevertheless, the different pattern of changes may allow a discrimination between both types of deposition layers. 

An additional information may be obtained in case of a thick (>1 mm thickness) hard deposition layer by considering the transmission time of a direct wave pulse between exciting and receiving transducer [31]. This approach, for which the velocity of sound of the layer material has to be known, can be realized with the present system by changing the frequency to the resonance frequency of a thickness vibration of the exciting transducer. In case of the single phase transducers used in the presence study, such a resonance occurs at a frequency of 1.875 MHz, where Lamb-type waves are hardly any longer excited due to the mismatch between the electrode structure of the transducer and the wavelength on the tube at that frequency. Thus a frequency switch would allow a unique discrimination between different types of deposition layers without any need of changing the set-up.

### 4.3. Filling Level Measurements in Horizontal Tubes

**Figure 17:**
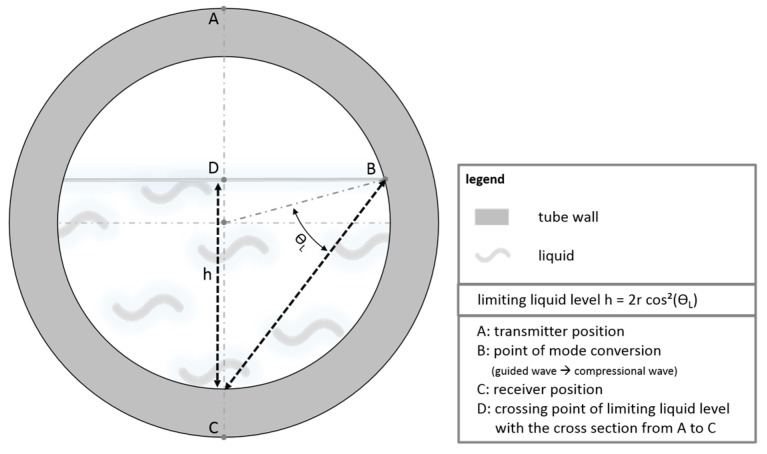
Explanation of pathways of sound waves crossing the liquid in a partially filled horizontal tube.

The filling level in horizontal tubes can also be determined by switching the transducer to a thickness vibration resonance and measure the echo time in a pulse-echo mode as described in Section 3.4. In addition, the Lamb-type wave excited by the upper transducer at a frequency of 1 MHz can be employed as well for the surveillance of a certain filling level, because only above this level a mode-converted compressional wave across the water starting from the tube wall at B will be able to arrive at the lower transducer C and to produce a corresponding transducer signal, as explained by Figure 17.

## 5. Conclusions

Using gelatin layers as a proxy for soft deposit layers inside water-filled steel tubes it is demonstrated that detection of the formation of such layers and of their thickness is possible with circumferential Lamb-type waves excited and detected on the outer wall of the tube with piezoelectric single-phase transducers operated at a frequency of 1 MHz and attached by a clamp-on device. Both the leaky circumferential guided waves travelling along the wall and the mode-converted wave crossing the water filling are represented in the receiver signal in different time regimes and their respective shifts of transmission time and attenuations are correlated with an increasing layer thickness. The change of both quantities allow a discrimination from temperature effects, which mainly cause time shifts alone. A tentative explanation for the unexpected large time shifts of the mode-converted wave is given in terms of a change of the emission angle due to the formation of a bilayer with a viscoelastic contact layer to water by the gelatin deposition on the steel wall and its influence on the circumferential guided wave mode. Additionally, it is shown that hard deposition layers can be detected and discriminated from soft layers and that the filling level in partly-filled horizontal tubes can be determined with the same set-up by switching the transducer frequency to a vibrational thickness resonance at 1.875 MHz.

## 6. Patents

An European patent application covering the method and the device described in the paper has been submitted and is under review [32].
